# Tracing the evolutionary history of hepatitis B virus genotype H endemic to Mexico

**DOI:** 10.3389/fmicb.2023.1180931

**Published:** 2023-05-24

**Authors:** Alexis Jose-Abrego, Sonia Roman, Saul Laguna-Meraz, João Renato Rebello-Pinho, Santiago Justo Arevalo, Arturo Panduro

**Affiliations:** ^1^Department of Genomic Medicine in Hepatology, Civil Hospital of Guadalajara, "Fray Antonio Alcalde", Guadalajara, Jalisco, Mexico; ^2^Health Sciences Center, University of Guadalajara, Guadalajara, Jalisco, Mexico; ^3^Molecular Biology in Medicine Doctorate Program, Guadalajara, Mexico; ^4^Department of Gastroenterology, Institute of Tropical Medicine and School of Medicine, LIM07, University of São Paulo, São Paulo, Brazil; ^5^Hospital Israelita Albert Einstein, São Paulo, Brazil; ^6^Faculty of Biological Sciences, Ricardo Palma University, Lima, Peru; ^7^Department of Biochemistry, Institute of Chemistry, University of São Paulo, São Paulo, Brazil

**Keywords:** hepatitis B virus, Mexico, origin, evolution, genotype H, Mesoamerica, indigenous populations

## Abstract

Hepatitis B virus (HBV) spreads efficiently among all human populations worldwide. HBV is classified into ten genotypes (A to J) with their geographic distribution and clinical features. In Mexico, HBV genotype H is the leading cause of hepatitis B and has been detected in indigenous populations, suggesting that HBV genotype H may be native to Mexico. However, little is known about the evolutionary history of HBV genotype H. Thus, we aimed to determine the age of HBV genotype H in Mexico using molecular dating techniques. Ninety-two HBV sequences of the reverse transcriptase (RT) domain of the polymerase gene (~1,251 bp) were analyzed; 48 were genotype H, 43 were genotype F, and the oldest HBV sequence from America was included as the root. All sequences were aligned, and the most recent common ancestor (TMRCA) time was calculated using the Bayesian Skyline Evolutionary Analysis. Our results estimate a TMRCA for the genotype H in Mexico of 2070.9 (667.5–4489.2) years before the present (YBP). We identified four major diversification events in genotype H, named H1, H2, H3, and H4. The TMRCA of H1 was 1213.0 (253.3–2638.3) YBP, followed by H2 1175.5 (557.5–2424.2) YBP, H3 949.6 (279.3–2105.0) YBP, and H4 1230.5 (336.3, 2756.7) YBP. We estimated that genotype H diverged from its sister genotype F around 8140.8 (1867.5–18012.8) YBP. In conclusion, this study found that genotype H in Mexico has an estimated age of 2070.9 (667.5–4489.2) YBP and has experienced at least four major diversification events since then.

## 1. Introduction

Tracing the evolutionary history of the hepatitis B virus (HBV) is essential for understanding its distribution and different degrees of adaptation to human populations (Roman et al., 2014). One of the most advanced forms of HBV adaptation is complete adaptation, where both the host and HBV genotype can coexist for extended periods due to a balance between the host’s immune response and viral replication (Roman et al., 2014). Probably, a complete adaptation would take thousands of years together to develop. Currently, 10 HBV genotypes have their geographic distribution (Velkov et al., 2018). HBV genotypes A and D are considered predominant in Europe, while genotypes C, B, and J are prevalent in Asian countries such as China, Korea, Japan, and Vietnam (Tian and Jia, 2016; Velkov et al., 2018). Genotype E is mainly detected in Africa and some Caribbean islands with African ancestry (Brichler et al., 2013). Genotype G has been associated mainly with men who have sex with men (MSM) and was reported initially in the United States and France. However, in recent years, genotype G has become one of the leading causes of hepatitis B in Mexico (Roman and Panduro, 2013).

On the other hand, the evolutionary analysis places genotypes F and H at the base of the phylogenetic tree, close to primate sequences such as the woolly monkey hepatitis B virus (Rasche et al., 2019). While genotype F is commonly detected in Central and South America (Alvarado-Mora and Pinho, 2013), its sister genotype H, is distributed in the north, center, and south of Mexico (Panduro et al., 2013). Genotype H has been found in several populations, including Mexican mestizos with Human Immunodeficiency Virus (HIV), hepatitis C patients, blood donors, and MSM, as well as in Nahuas and Huicholes, two indigenous groups in western Mexico (Roman et al., 2010; Alvarez-Munoz et al., 2014; Jose-Abrego et al., 2021). It has also been detected in the Oaxaca state, where 39.1% of the population speaks an indigenous language (INEGI, 1995; Jose-Abrego et al., 2017). Notably, a recent archaeological study found genotype H in a tooth dating back 522 years, recovered from the remains of the “Real de San José de los Naturales” hospital that mainly served the indigenous population during the colonial period (INAH, 2012; Kocher et al., 2021). In native Mexicans, HBV genotype H infections are typically asymptomatic with low viral loads (<2000 IU/mL), and liver damage is insignificant or undetectable (Panduro et al., 2013). Furthermore, hepatocellular carcinoma associated with genotype H is rare in Mexico (Gomez-Quiroz and Roman, 2022). These observations suggest that genotype H is not pathogenic for the Mexican population, likely due to a high degree of adaptation that developed over centuries of coexistence with the endemic host. Therefore, in this study, we aimed to determine the time of the most common ancestor (TMRCA) to trace the evolutionary history of HBV genotype H endemic to Mexico.

## 2. Materials and methods

### 2.1. HBV dataset

**Figure 1:**
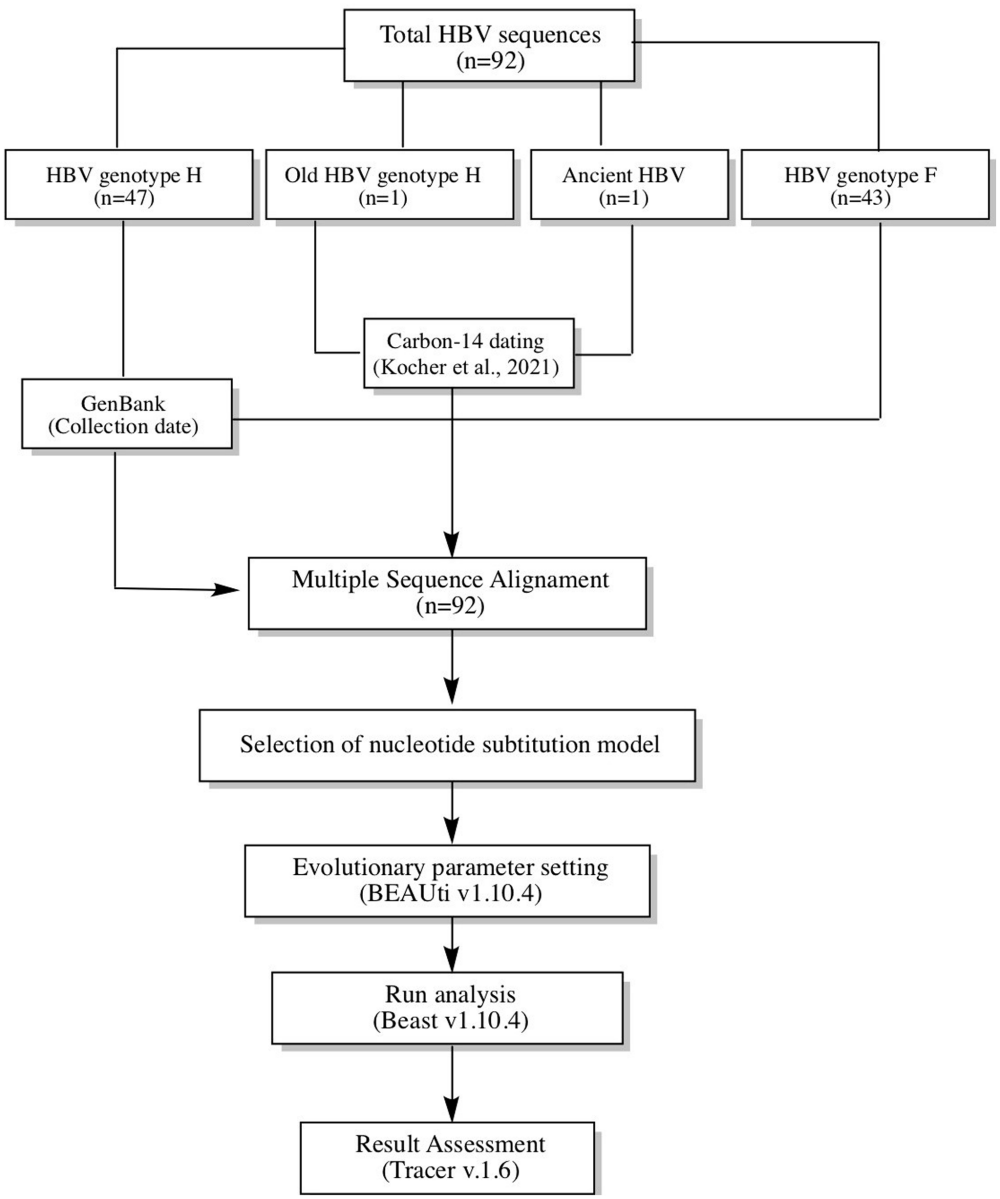
Workflow used to calculate the time to the Most Recent Common Ancestor (TMRCA) for genotype H. In total, 92 sequences were analyzed (48 genotype H and 43 genotype F). In addition, the oldest sequencing of hepatitis B in America was used as the root. All sequences were aligned and subjected to Bayesian coalescence analysis.

The study analyzed 92 HBV reverse transcriptase (RT) domain polymerase sequences (~1,251 bp), including 48 Mexican sequences of genotype H collected between 2005–2017 (one of which had 522 years of antiquity), 43 F1-F4 sequences collected between 1999–2017 to estimate the time genotype H diverged from F, and America’s oldest reported HBV sequence (CUN002) was used as the root (Kocher et al., 2021). Genetic information and data collection of the contemporary sequences were obtained from GenBank, while information on the ancestral HBV sequences was obtained from the publication of Kocher et al. (2021). Figure 1 provides a summary of the method used in the study.

### 2.2. Phylogenetic and Bayesian coalescent analysis

Before the Bayesian analysis, all HBV sequences were aligned with the ClustalW algorithm, and each HBV genotype was confirmed by phylogenetic analysis using HBV reference sequences from GenBank. These tests were carried out in Molecular Evolutionary Genetic Analysis Software v7.0.26. Next, the alignment was analyzed in the Jmodeltest v2.1.10 to select the nucleotide substitution model. Each HBV sequence was named with its country of origin, accession number, HBV genotype/sub-genotype, and data collection or archeological dating. The General Time Reversible (GTR) model was used as the nucleotide substitution model with an estimated base frequency. The site heterogeneity model was Gamma + Invariant Sites with four gamma categories. Coalescent Bayesian Skyline analysis was carried out using a strict and relaxed molecular clock. These settings were configured in BEAUti v1.10.4 software and ran in Beast Software v1.10.4. Bioinformatic experiments started with 10 million iterations and gradually increased until reaching values >200 Effective Sample Size (ESS) for each evolutionary parameter. Bayesian Skyline Reconstruction in Tracer v.1.6 software was used to infer changes in the number of HBV infections over time. Marginal likelihood values were compared using Akaike information criteria (AIC) with a bootstrap of 1,000 replicates. Lower AIC values suggested a better model fit. The tree of the best model fit was built with the maximum clade credibility (MCC) method with Tree-Annotator V1.10.4 software, and the time of the most recent common ancestor (TMRCA) was expressed as mean and 95% Highest Posterior Density (HPD).

None of the HBV sequences from this study showed evidence of recombination, this was evaluated with the hepatitis B Virus phylogenetic typing tool available at https://www.genomedetective.com/app/typingtool/hbv/.

## 3. Results

Four hundred million iterations were needed so that the evolutionary parameters achieved a mean value of 860 ESS (Table 1). Based on the AIC value, the relaxed model fitted better than the strict model (12827.9 vs. 12940.78). With this molecular clock, a mean evolutionary rate of 8.2 ×10^−6^ (95% HPD: 2.2×10^−6^ -1.5×10^−5^) substitutions/site/year for the RT domain of the polymerase gene was calculated.

**Table 1:** Estimated time to the Most Recent Common Ancestor (TMRCA) for genotypes H and F.

HBV cluster	TMRCA	95% HPD Interval	ESS
Root	15439.6	[9021.6, 31058.7]	901
F/H	8140.8	[1867.5, 18012.8]	901
H (Root)	2070.9	[667.5, 4489.2]	901
H1	1,213	[253.3, 2638.3]	800
H2	1175.5	[557.5, 2424.2]	901
H3	949.6	[279.3, 2105.0]	901
H4	1230.5	[336.3, 2756.7]	901
F (Root)	4551.9	[1311.1, 10031.9]	901
F1a	402.2	[8.7, 1141.1]	901
F1b	1269.1	[397.6, 3009.6]	901
F2a	987.2	[220.6, 2101.5]	901
F2b	856.5	[194.4, 1968.2]	901
F3	908.3	[106.7, 2182.8]	901
F4	1,164	[266.2, 2670.6]	901

HPD, Highest Posterior Density; ESS, Effective Sample Size.

**Figure 2:**
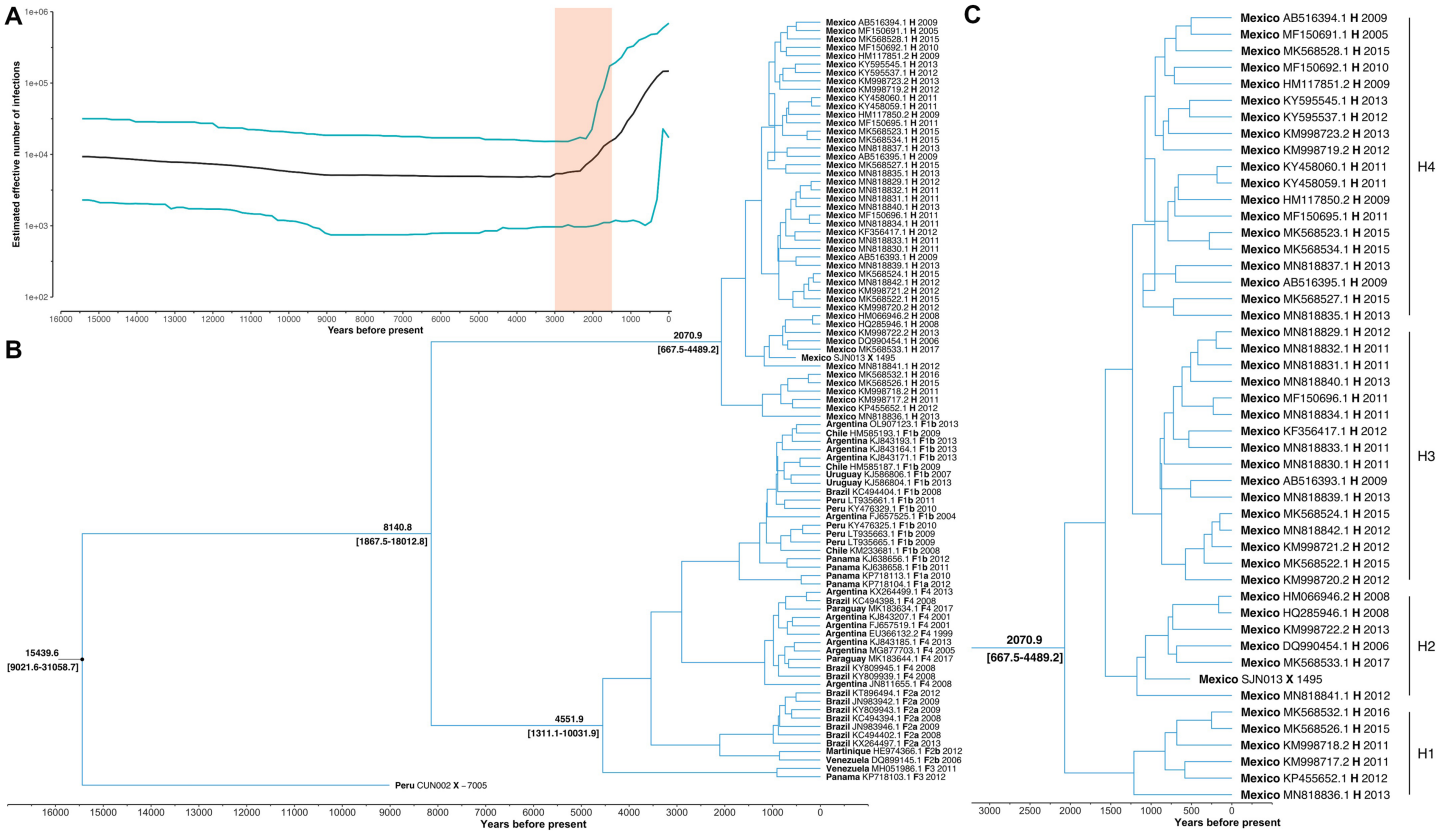
Main results of the Bayesian skyline analysis. **(A)** Reconstruction of HBV infections in the last 15,000 years. T = 0 indicates the year 2017, which was the most recent collection date. While the shaded area highlights a period of rapid growth in the number of HBV infections. **(B)** Phylogenetic tree showing the evolutionary history of genotype H. Values at nodes indicate the time of Most Recent Common Ancestor (TMRCA). Each sample is named with its country of origin, ID access, genotype/sub-genotype, and collection date. **(C)** The TMRCA of the genotype H and values in brackets represent 95% highest posterior density interval of the estimation. Letters (H1-H4) indicate the main diversification events of genotype H since its origin in Mexico.

The Bayesian Skyline reconstruction showed that from 15,500 to 3,000 YBP, the number of HBV infections remained stable at ~10,000 cases. However, during the period >3,000 to 1,500, there was a rapid increase in HBV infections (Figure 2A). According to our results, the TMRCA for the pre-genotype F/H was 15439.6 (95% HPD: 9021.6–31058.7) years before the present (YBP) (Figure 2B and Table 1). The speciation event between genotype H and F occurred 8140.8 (95% HPD: 1867.5–18012.8) YBP. After that, genotype F began its process of diversification into the four major sub-genotypes (F1-F4) 4551.9 (1311.1–1031.9) YBP (Table 1).

The TMRCA for Mexican genotype H sequences was 2070.9 (667–4489.2) YBP (Figure 2C). Considering an average human generation time of 26.9 years (Wang et al., 2023), we calculated that genotype H has accompanied Mexico’s native populations for at least 77 generations (2070.9 years x 1 human generation/26.9 years). During this period, four clusters were identified (H1, H2, H3, and H4). TMRCA of H1 was 1213.0 (253.3–2638.3) YBP, followed by H2 1175.5(557.5–2424.2) YBP, H3 949.6 (279.3–2105.0) YBP, and H4 1230.5 (336.3, 2756.7) YBP. Interestingly, the ancient Mexican genotype H (SJN013) was grouped in H2 (Figure 2C).

## 4. Discussion

The HBV genotype H is more prevalent in Mexico than in any other region in the world (Roman et al., 2014). Clinical evidence suggests that genotype H has developed a high degree of adaptation with the Mexican population, particularly with native populations (Roman et al., 2014). Tracing the evolutionary history of genotype H can provide insight into its origin, spread, and evolution within human populations.

**Figure 3:**
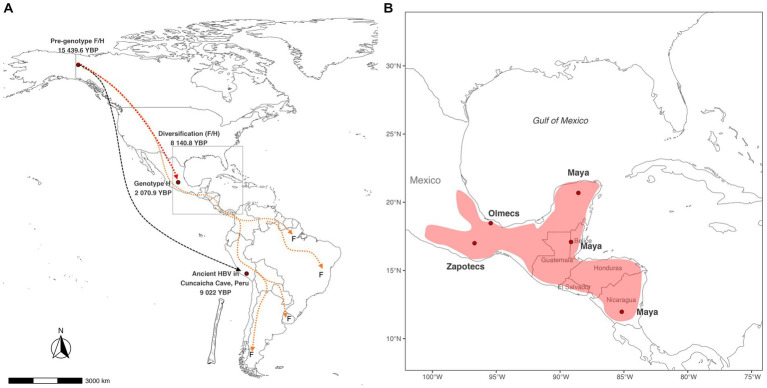
Potential routes of HBV spread in the Americas. **(A)** The pre-genotype F/H arrived in North America 15,439.6 Years Before Present (YBP) and split into two main lineages. Archaeological evidence suggests that the first HBV lineage reached the highlands of Peru at 9022 YBP. At the same time, the second lineage originated the genotypes F and H 8140 YBP. Populations carrying the genotype H remained in Mexico, while the genotype F carriers continued to travel until reaching South America. **(B)** Theoretical distribution of the genotype H considering the main cultures in Mesoamerica when the genotype H emerged 2070.9 YBP.

Based on our results, we calculated that the pre-genotype F/H arrived in the Americas at 15,439.6 (9021–31058.7) YBP (Figure 3A); this time correlates with the last deglaciation of North America (21000–10,000 YBP) (Lora and Ibarra, 2019). The pre-genotype F/H could have emerged in Alaska-Yukon settlements that used the Coastal Route or Ice-free corridor to reach South America 19,000–15,500 years ago (Lesnek et al., 2018). This finding would explain why some cases of genotype F have been found in Alaska’s indigenous populations (Livingston et al., 2007). Currently, the pre-genotype F/H is extinct, and the evidence supporting or rejecting this hypothesis could be found in the genetic information of human remains from the North American region. According to our results, the pre-genotype F/H has remained genetically stable until the split in H and F 8140.8 (1867.5–18012.8) YBP. This time correlates with domestication of *Cucurbita pepo* (pumpkins) by the inhabitants of Oaxaca’s highlands of Mexico between 9,000–7,000 carbon-14 YBP (Smith, 1997). This finding suggests that the diversification of genotypes H and F could have occurred during the onset of plant domestication and the development of early societies among indigenous peoples (Smith, 2006; Piperno, 2011). After the division into the F and H lineages, genotype F began its diversification 2,500 years before genotype H, indicating that the TMRCA of genotype F is older than the TMRCA of genotype H. This might be explained by several reasons, one hypothesis is that the genotype F could have emerged in the first waves of human migration (Waters, 2019), while the genotype H emerged in a more recent migration. It is also possible that carriers of the genotype F may have taken faster migratory routes (Stoneking et al., 2023) than ancestors of the genotype H, leading them to experience a variety of environmental conditions that impacted the rate of evolution of the genotype F (Rasche et al., 2019; Lindo and Degiorgio, 2021). This factor together with geographic isolation could have originated the current F sub-genotypes (Mojsiejczuk et al., 2020). On the other hand, the civilizations carrying the genotype H prospered in Mexico and Central America, developing plant domestication, agriculture and building the city-states of Mesoamerica. In overall, the factors that contributed to the divergence of the two genotypes are complex and may include differences in migration routes, timing of migrations, and environmental conditions. It is important to continue studying the evolutionary history of HBV in the Americas to gain a better understanding of the genetic diversity of this virus and its impact on human health in the region.

On the other hand, the population reconstruction analysis identified an increase in HBV infections during the period >3,000 to 1,500 YBP. Explaining this event is difficult due to limited historical information. However, several factors may have contributed to the spread of HBV infection in Mesoamerican societies. One possibility could be that the increase in population density in the city-states (Posth et al., 2018) led to more opportunities for HBV transmission through sexual contact. Additionally, large armies, often composed of men, would have increased the risk of HBV transmission in villages submitted by the Aztecs during the postclassic period (900–1,519 AD) (Garraty, 2006; Pennock, 2022). Another possible factor is that many cultures practiced human sacrifice, anthropophagy, and rituals that involved sexual activity (De Montellano, 1978; Evans, 1998). The increase in the number of HBV infections could also be influenced by the appearance of the current genotype H. Our molecular dating suggests that the genotype H emerged in Mexico 2070.9 (667.5–4489.2) YBP. This time is within the range published by Paraskevis et al. (2013), who dated the age of the genotype H to 4,100 (1500–7,100) YBP. It was also consistent with the results of Kocher et al., in 2021. However, it differs from that reported by Wolf et al. (2021), who calculated an age for the genotype H of 87 YBP. However, their analysis described the contemporary spread of genotype H to other countries. The differences with that study may be due to the limited availability of genotype H sequences that existed at that time. Here, we included calibration points resulting from anthropological studies with carbon-14 and maximized the number of genotype H sequences using the RT domain of the polymerase gene. Converting 2,070.9 years to human generations, we estimate that HBV genotype H has accompanied the Mexican population for 77 generations, which could be necessary for genotype H to develop its high degree of adaptation with the indigenous population of Mexico. In addition, the TMRCA of genotype H coincides with the pre-classic period (2000 BC - 250 AD) in Mesoamerica (Figure 3B), when powerful civilizations such as the Olmecs in Veracruz, the Zapotecs in Oaxaca, and the Mayas in southern Mexico, Guatemala, Honduras, and northern Nicaragua appeared (Guernsey, 2010). This hypothetical distribution could explain why some cases of genotype H were found in Nicaragua (Arauz-Ruiz et al., 2002). Here, we report that genotype H has presented four major diversification events, denoted as H1, H2, H3, and H4. Most of these clusters had an average TMRCA >1,000 YBP. Interestingly, the ancestral genotype H (SNN013) was classified in group H2, suggesting that the virus’s descendants may circulate in Mexican patients. Future whole-genome studies are needed to confirm whether these clusters may be considered new H subgenotypes. In this study, it is proposed that the genotype H is endemic to Mexico and nearby regions to Mesoamerica. This hypothesis is supported by the high prevalence of genotype H in Mexico (Panduro et al., 2013). Additionally, the genotype H has been identified in native populations of Mexico (Roman et al., 2010; Jose-Abrego et al., 2021), suggesting that it has been present in the region for a long time. Furthermore, it has been observed that the genotype H in Mexican Amerindian populations is less pathogenic, suggesting a high degree of adaptation between the virus and its host (Roman et al., 2014; Jose-Abrego et al., 2017). Notably, the identification of the genotype H sequence in human remains from Mexico (Kocher et al., 2021) adds weight to the idea that the virus has been present in the region for thousands of years. Here, we estimate that the genotype H has accompanied the Mexican population for at least 2070.9 years. However, in the future, with the new discovery of HBV in human remains in Mexico, particularly in the Yucatan peninsula (Macdonald et al., 2020), we do not rule out the possibility that genotype H may be even older than expected.

The results of this study correlate with the prehistoric migrations proposed by the Out-of-Africa hypothesis (Locarnini et al., 2021). However, due to the complex evolutionary history of HBV, it is likely that current or extinct HBV genotypes may have more than one pathway to explain their origin (Souza et al., 2014). For example, it has been proposed that recombination of the old HBV genotypes could originate the current genotype A in Europe (Muhlemann et al., 2018). Similarly, genotype I could result from the recombination of the sub-genotype C2 and two extinct genotypes (Datta, 2020). On the other hand, the American HBV genotypes (F and H) are the most distant from the rest of the human genotypes and are related to HBV detected in New World Monkeys (Norder et al., 2004; De Carvalho Dominguez Souza et al., 2018). Due to their genetic similarities, it is suggested that the ancestor of genotypes F/H could have emerged in New World primates that arrived through transoceanic migration millions of years ago (Datta, 2020). This ancestor could have been able to jump to humans during the early colonization of the Americas. In addition, in 2013, five HBV-related sequences were identified in bats from Panama, the authors concluded that bats could be ancestral sources of primate *hepadnaviruses* (Rasche et al., 2016). These findings suggest that the evolutionary history of genotypes F and H is very long and complex. Further studies are needed to confirm this hypothesis and to explore the possible role of other animal species, such as monkeys and bats, in the evolution of HBV.

Our study has several limitations that need to be considered when interpreting the results. Firstly, most HBV genotype H sequences were from western Mexico which may not be representative of the entire population. Second, in order to maximize the sample size, this study focused on the RT domain of the polymerase gene that could explain only part of whole evolutionary history of HBV genotype H. Third, as with all studies based on Bayesian phylogenetic inference methods, several factors can affect the molecular dating and its 95% HPD interval, including heterochrony calibration points, sample size, choice of molecular markers, and fit nucleotide substitution model (Ho et al., 2011). Despite these limitations, our study provides valuable information about the evolutionary history of HBV genotype H in Mexico. It is important to note that this study is the largest to date that attempts to reconstruct the evolution and diversification of HBV genotype H. Our results support the hypothesis that the HBV genotype H is endemic to Mexico, particularly to the Mesoamerican region. Our study proposes the number of human generations required for the genotype H to develop its high degree of adaptation with native populations. Finally, this study highlights the importance of analyzing the virome of human remains and indigenous populations to elucidate the complete history of HBV genotype H and its relationship with other genotypes in the American continent.

In conclusion, this study traced the evolutionary history of genotype H from a Mesoamerican historical approach. The molecular data analysis revealed an estimated TMRCA of 2070.9 (667.5–4489.2) YBP coinciding with the development of the ancient Mesoamerican societies, highly spreading throughout the population between >3,000–1,500 YBP and experiencing at least four major diversification events since its emergence in Mexico.

## Data availability statement

The datasets presented in this study can be found in online repositories. The names of the repository/repositories and accession number(s) can be found in the article/Supplementary material.

## Author contributions

AP and SR: conceptualization. AP, SR, JR-P, and SJA: methodology. SJA: validation. AJ-A, SR, and AP: formal analysis and investigation. AJ-A: data curation. AJ-A: writing-original draft preparation. SR, AP, and JR-P: writing-review and editing. SR: supervision. AP: funding acquisition. All authors contributed to the article and approved the submitted version.

## Funding

This research was partly funded by Consejo Nacional de Ciencia y Tecnología (CONACYT), Grant Number CONACYT PN-2017-01-5254 to AP and Programa de Apoyo a la Incorporación de NPTC. no. UDG-PTC-1439 to AJ-A. This work was part of AJ-A’s thesis to obtain the degree in Doctor of Science in Molecular Biology in Medicine, University of Guadalajara.

## Conflict of interest

The authors declare that the research was conducted in the absence of any commercial or financial relationships that could be construed as a potential conflict of interest.

## Publisher’s note

All claims expressed in this article are solely those of the authors and do not necessarily represent those of their affiliated organizations, or those of the publisher, the editors and the reviewers. Any product that may be evaluated in this article, or claim that may be made by its manufacturer, is not guaranteed or endorsed by the publisher.
