# Supplementary material for: Tracing the evolutionary history of hepatitis B virus genotype H endemic to Mexico
Source: Front Microbiol. 2023 May 24;14:1180931. doi: 10.3389/fmicb.2023.1180931 (PMC10244555; doi:10.3389/fmicb.2023.1180931)
Supplement: Supplementary file 1 [file Table_1.docx]

| Table supplementary 1. Accession numbers of HBV sequences used in this study | | | | | | |
| --- | --- | --- | --- | --- | --- | --- |
| n | Accession numbers | HBV Genotype | Country | Date | Data Base | Tree label |
| 1 | MN818830.1 | H | Mexico | 2011 | Nucleotide | Mexico_MN818830.1_H_2011 |
| 2 | MN818840.1 | H | Mexico | 2013 | Nucleotide | Mexico_MN818840.1_H_2013 |
| 3 | MN818832.1 | H | Mexico | 2011 | Nucleotide | Mexico_MN818832.1_H_2011 |
| 4 | MF150696.1 | H | Mexico | 2011 | Nucleotide | Mexico_MF150696.1_H_2011 |
| 5 | MN818834.1 | H | Mexico | 2011 | Nucleotide | Mexico_MN818834.1_H_2011 |
| 6 | MN818833.1 | H | Mexico | 2011 | Nucleotide | Mexico_MN818833.1_H_2011 |
| 7 | MN818831.1 | H | Mexico | 2011 | Nucleotide | Mexico_MN818831.1_H_2011 |
| 8 | MN818829.1 | H | Mexico | 2012 | Nucleotide | Mexico_MN818829.1_H_2012 |
| 9 | KF356417.1 | H | Mexico | 2012 | Nucleotide | Mexico_KF356417.1_H_2012 |
| 10 | AB516393.1 | H | Mexico | 2009 | Nucleotide | Mexico_AB516393.1_H_2009 |
| 11 | MN818839.1 | H | Mexico | 2013 | Nucleotide | Mexico_MN818839.1_H_2013 |
| 12 | KY458059.1 | H | Mexico | 2011 | Nucleotide | Mexico_KY458059.1_H_2011 |
| 13 | KY458060.1 | H | Mexico | 2011 | Nucleotide | Mexico_KY458060.1_H_2011 |
| 14 | HM117850.2 | H | Mexico | 2009 | Nucleotide | Mexico_HM117850.2_H_2009 |
| 15 | MK568522.1 | H | Mexico | 2015 | Nucleotide | Mexico_MK568522.1_H_2015 |
| 16 | MK568524.1 | H | Mexico | 2015 | Nucleotide | Mexico_MK568524.1_H_2015 |
| 17 | MN818842.1 | H | Mexico | 2012 | Nucleotide | Mexico_MN818842.1_H_2012 |
| 18 | KM998720.2 | H | Mexico | 2012 | Nucleotide | Mexico_KM998720.2_H_2012 |
| 19 | KM998721.2 | H | Mexico | 2012 | Nucleotide | Mexico_KM998721.2_H_2012 |
| 20 | AB516395.1 | H | Mexico | 2009 | Nucleotide | Mexico_AB516395.1_H_2009 |
| 21 | MN818837.1 | H | Mexico | 2013 | Nucleotide | Mexico_MN818837.1_H_2013 |
| 22 | MK568523.1 | H | Mexico | 2015 | Nucleotide | Mexico_MK568523.1_H_2015 |
| 23 | MK568534.1 | H | Mexico | 2015 | Nucleotide | Mexico_MK568534.1_H_2015 |
| 24 | MN818835.1 | H | Mexico | 2013 | Nucleotide | Mexico_MN818835.1_H_2013 |
| 25 | MK568527.1 | H | Mexico | 2015 | Nucleotide | Mexico_MK568527.1_H_2015 |
| 26 | MF150695.1 | H | Mexico | 2011 | Nucleotide | Mexico_MF150695.1_H_2011 |
| 27 | MK568528.1 | H | Mexico | 2015 | Nucleotide | Mexico_MK568528.1_H_2015 |
| 28 | AB516394.1 | H | Mexico | 2009 | Nucleotide | Mexico_AB516394.1_H_2009 |
| 29 | HM117851.2 | H | Mexico | 2009 | Nucleotide | Mexico_HM117851.2_H_2009 |
| 30 | MF150691.1 | H | Mexico | 2005 | Nucleotide | Mexico_MF150691.1_H_2005 |
| 31 | MF150692.1 | H | Mexico | 2010 | Nucleotide | Mexico_MF150692.1_H_2010 |
| 32 | KY595537.1 | H | Mexico | 2012 | Nucleotide | Mexico_KY595537.1_H_2012 |
| 33 | KY595545.1 | H | Mexico | 2013 | Nucleotide | Mexico_KY595545.1_H_2013 |
| 34 | KM998723.2 | H | Mexico | 2013 | Nucleotide | Mexico_KM998723.2_H_2013 |
| 35 | KM998719.2 | H | Mexico | 2012 | Nucleotide | Mexico_KM998719.2_H_2012 |
| 36 | HM066946.2 | H | Mexico | 2008 | Nucleotide | Mexico_HM066946.2_H_2008 |
| 37 | HQ285946.1 | H | Mexico | 2008 | Nucleotide | Mexico_HQ285946.1_H_2008 |
| 38 | KM998722.2 | H | Mexico | 2013 | Nucleotide | Mexico_KM998722.2_H_2013 |
| 39 | SJN013 | X | Mexico | 1495 | BioSample | Mexico_SJN013_X_1495 |
| 40 | MK568533.1 | H | Mexico | 2017 | Nucleotide | Mexico_MK568533.1_H_2017 |
| 41 | DQ990454.1 | H | Mexico | 2006 | Nucleotide | Mexico_DQ990454.1_H_2006 |
| 42 | MN818841.1 | H | Mexico | 2012 | Nucleotide | Mexico_MN818841.1_H_2012 |
| 43 | MK568526.1 | H | Mexico | 2015 | Nucleotide | Mexico_MK568526.1_H_2015 |
| 44 | MK568532.1 | H | Mexico | 2016 | Nucleotide | Mexico_MK568532.1_H_2016 |
| 45 | KM998717.2 | H | Mexico | 2011 | Nucleotide | Mexico_KM998717.2_H_2011 |
| 46 | KP455652.1 | H | Mexico | 2012 | Nucleotide | Mexico_KP455652.1_H_2012 |
| 47 | KM998718.2 | H | Mexico | 2011 | Nucleotide | Mexico_KM998718.2_H_2011 |
| 48 | MN818836.1 | H | Mexico | 2013 | Nucleotide | Mexico_MN818836.1_H_2013 |
| 49 | KC494398.1 | F4 | Brazil | 2008 | Nucleotide | Brazil_KC494398.1_F4_2008 |
| 50 | KX264499.1 | F4 | Argentina | 2013 | Nucleotide | Argentina_KX264499.1_F4_2013 |
| 51 | KJ843207.1 | F4 | Argentina | 2001 | Nucleotide | Argentina_KJ843207.1_F4_2001 |
| 52 | FJ657519.1 | F4 | Argentina | 2001 | Nucleotide | Argentina_FJ657519.1_F4_2001 |
| 53 | JN811655.1 | F4 | Argentina | 2008 | Nucleotide | Argentina_JN811655.1_F4_2008 |
| 54 | EU366132.2 | F4 | Argentina | 1999 | Nucleotide | Argentina_EU366132.2_F4_1999 |
| 55 | MK183634.1 | F4 | Paraguay | 2017 | Nucleotide | Paraguay_MK183634.1_F4_2017 |
| 56 | MK183644.1 | F4 | Paraguay | 2017 | Nucleotide | Paraguay_MK183644.1_F4_2017 |
| 57 | KY809945.1 | F4 | Brazil | 2008 | Nucleotide | Brazil_KY809945.1_F4_2008 |
| 58 | MG877703.1 | F4 | Argentina | 2005 | Nucleotide | Argentina_MG877703.1_F4_2005 |
| 59 | KJ843185.1 | F4 | Argentina | 2013 | Nucleotide | Argentina_KJ843185.1_F4_2013 |
| 60 | KY809939.1 | F4 | Brazil | 2008 | Nucleotide | Brazil_KY809939.1_F4_2008 |
| 61 | JN983942.1 | F2a | Brazil | 2009 | Nucleotide | Brazil_JN983942.1_F2a_2009 |
| 62 | KT896494.1 | F2a | Brazil | 2012 | Nucleotide | Brazil_KT896494.1_F2a_2012 |
| 63 | KC494394.1 | F2a | Brazil | 2008 | Nucleotide | Brazil_KC494394.1_F2a_2008 |
| 64 | KY809943.1 | F2a | Brazil | 2009 | Nucleotide | Brazil_KY809943.1_F2a_2009 |
| 65 | KC494402.1 | F2a | Brazil | 2008 | Nucleotide | Brazil_KC494402.1_F2a_2008 |
| 66 | JN983946.1 | F2a | Brazil | 2009 | Nucleotide | Brazil_JN983946.1_F2a_2009 |
| 67 | KX264497.1 | F2a | Brazil | 2013 | Nucleotide | Brazil_KX264497.1_F2a_2013 |
| 68 | HE974366.1 | F2b | Martinique | 2012 | Nucleotide | Martinique_HE974366.1_F2b_2012 |
| 69 | DQ899145.1 | F2b | Venezuela | 2006 | Nucleotide | Venezuela_DQ899145.1_F2b_2006 |
| 70 | KJ843193.1 | F1b | Argentina | 2013 | Nucleotide | Argentina_KJ843193.1_F1b_2013 |
| 71 | OL907123.1 | F1b | Argentina | 2013 | Nucleotide | Argentina_OL907123.1_F1b_2013 |
| 72 | HM585193.1 | F1b | Chile | 2009 | Nucleotide | Chile_HM585193.1_F1b_2009 |
| 73 | KJ843164.1 | F1b | Argentina | 2013 | Nucleotide | Argentina_KJ843164.1_F1b_2013 |
| 74 | KJ843171.1 | F1b | Argentina | 2013 | Nucleotide | Argentina_KJ843171.1_F1b_2013 |
| 75 | HM585187.1 | F1b | Chile | 2009 | Nucleotide | Chile_HM585187.1_F1b_2009 |
| 76 | KJ586806.1 | F1b | Uruguay | 2007 | Nucleotide | Uruguay_KJ586806.1_F1b_2007 |
| 77 | KJ586804.1 | F1b | Uruguay | 2013 | Nucleotide | Uruguay_KJ586804.1_F1b_2013 |
| 78 | KC494404.1 | F1b | Brazil | 2008 | Nucleotide | Brazil_KC494404.1_F1b_2008 |
| 79 | FJ657525.1 | F1b | Argentina | 2004 | Nucleotide | Argentina_FJ657525.1_F1b_2004 |
| 80 | KY476329.1 | F1b | Peru | 2010 | Nucleotide | Peru_KY476329.1_F1b_2010 |
| 81 | KY476325.1 | F1b | Peru | 2010 | Nucleotide | Peru_KY476325.1_F1b_2010 |
| 82 | LT935663.1 | F1b | Peru | 2009 | Nucleotide | Peru_LT935663.1_F1b_2009 |
| 83 | LT935665.1 | F1b | Peru | 2009 | Nucleotide | Peru_LT935665.1_F1b_2009 |
| 84 | LT935661.1 | F1b | Peru | 2011 | Nucleotide | Peru_LT935661.1_F1b_2011 |
| 85 | KM233681.1 | F1b | Chile | 2008 | Nucleotide | Chile_KM233681.1_F1b_2008 |
| 86 | KJ638658.1 | F1b | Panama | 2011 | Nucleotide | Panama_KJ638658.1_F1b_2011 |
| 87 | KJ638656.1 | F1b | Panama | 2012 | Nucleotide | Panama_KJ638656.1_F1b_2012 |
| 88 | KP718104.1 | F1a | Panama | 2012 | Nucleotide | Panama_KP718104.1_F1a_2012 |
| 89 | KP718113.1 | F1a | Panama | 2010 | Nucleotide | Panama_KP718113.1_F1a_2010 |
| 90 | MH051986.1 | F3 | Venezuela | 2011 | Nucleotide | Venezuela_MH051986.1_F3_2011 |
| 91 | KP718103.1 | F3 | Panama | 2012 | Nucleotide | Panama_KP718103.1_F3_2012 |
| 92 | CUN002 | X | Peru | 9022 | BioSample | Peru_CUN002_X_9022 |
| Source: National Center for Biotechnology information, https://www.ncbi.nlm.nih.gov/biosample/?term=CUN002 | | | | | | |
